# Joint Power Charging and Routing in Wireless Rechargeable Sensor Networks

**DOI:** 10.3390/s17102290

**Published:** 2017-10-09

**Authors:** Jie Jia, Jian Chen, Yansha Deng, Xingwei Wang, Abdol-Hamid Aghvami

**Affiliations:** 1 Key Laboratory of Medical Image Computing of Northeastern University, Ministry of Education, Shenyang 110819, China; jiajie@mail.neu.edu.cn (J.J.); wangxingwei@mail.neu.edu.cn (X.W.); 2 School of Computer Science and Engineering, Northeastern University, Shenyang 110819, China; 3 Department of Informatics, King’s College London, London WC2R 2LS, UK; yansha.deng@kcl.ac.uk (Y.D.); hamid.aghvami@kcl.ac.uk (A.-H.A.)

**Keywords:** WRSNs, charging efficiency, routing, GA, heuristic algorithm

## Abstract

The development of wireless power transfer (WPT) technology has inspired the transition from traditional battery-based wireless sensor networks (WSNs) towards wireless rechargeable sensor networks (WRSNs). While extensive efforts have been made to improve charging efficiency, little has been done for routing optimization. In this work, we present a joint optimization model to maximize both charging efficiency and routing structure. By analyzing the structure of the optimization model, we first decompose the problem and propose a heuristic algorithm to find the optimal charging efficiency for the predefined routing tree. Furthermore, by coding the many-to-one communication topology as an individual, we further propose to apply a genetic algorithm (GA) for the joint optimization of both routing and charging. The genetic operations, including tree-based recombination and mutation, are proposed to obtain a fast convergence. Our simulation results show that the heuristic algorithm reduces the number of resident locations and the total moving distance. We also show that our proposed algorithm achieves a higher charging efficiency compared with existing algorithms.

## 1. Introduction

Because of the wide applications of wireless sensor networks (WSNs) in environmental monitoring, ecosystem surveillance, and physical hazard prevention, WSNs have attracted a flourish of research efforts from both the industry and academia in the last decade. However, most WSNs still face a limited operating time, as they are powered by battery. In order to prolong the network lifetime, extensive research efforts have been focused on low-power hardware architecture [1], low-complexity software implementation [2], power-efficient wireless communication [3], topology control [4], and dynamic routing techniques [5]. Although these solutions can extend the network lifetime to some extent, the limited battery remains a paramount hurdle to the development of large-scale applications. Some other solutions have resorted to energy harvesting sources, such as solar, wind, hydroelectric, and thermoelectric. However, these harvesting sources are generally expensive and with a large size, requiring an additional energy harvesting component, and they may not always be accessible because of the environment dynamics [6]. For example, the availability of a solar-based harvesting system drastically varies with time and weather, and these cannot provide enough energy for duty-cycle applications [7].

In place of conventional energy harvesting techniques, the recent breakthrough in the area of wireless power transfer (WPT) has opened up a promising alternative to power devices using radio frequency (RF) signals over the air. It was shown in an Intel research report that a wireless identification and sensing platform (WISP) can harvest energy to power the operation of micro-controller unit (MCU) [8]. In their study, WPT was performed by harvesting energy from the ambient RF signals radiated by the surrounding transmitters, and they also showed that WPT technology is fully adjustable in its transmit power, waveforms, and resource blocks. Regarding the energy harvesting efficiency, the results in [9] showed that nearly 40 μW can be transferred over a distance of 10 m with a transmit power of 3 W, which is sufficient to power the operations of many low-power devices, such as sensors and RF identification (RFID) tags. As a result of the inherent energy harvesting characteristic of the WPT technique, a relatively short transmission distance and multiple simultaneous transmissions in WSNs, wireless rechargeable sensor networks (WRSNs) can be a promising solution to achieve high energy efficiency, and they have attracted much attention recently [10].

In WRSNs, the rechargeable sensors are capable of harvesting energy from the RF signals transmitted by energy sources to power the data sensing from the environment and data delivered to the sink. In most applications, these energy sources are mobile chargers carried by autonomous vehicles [11] to tackle the dynamic topology problem. With the moving speed and the predefined charging capacity, the modeling, characterization and optimization of charging in WRSNs becomes extremely important. There has been some research focusing on charging optimization in WRSNs. In [12], by importing the concept of a renewable energy cycle, an optimal traveling path for a mobile charging vehicle is designed. In [13], velocity control with a predefined charging trajectory is designed to satisfy the traveling time constraint. In [14], as a result of the redundant deployment of sensors, the optimal energy replenishment joint with coverage control is designed to achieve the balance between the coverage rate and the energy partitioning. However, all these works assume that the data-gathering tree is predefined before energy harvesting. In [15], a joint routing and charging schedule was proposed to prolong the network lifetime. For cluster-based WSNs, an energy-efficient cooperative transmission optimization was proposed in [16], for which the power allocation, power splitting and relay selection were formulated as a non-convex problem, and a distributed iteration algorithm based on fractional programming and dual decomposition was proposed. However, the charging model defined in their work can harvest energy to only one sensor each time, which may cause a longer charging time for large-scale applications. Assuming that the mobile charger can also be a data collector, the joint mobile energy replenishment and data gathering are considered [17,18,19]. However, because of downlink (DL) WPT and uplink (UL) information transmission, the energy replenishment and data gathering should work in different time slots, which may induce larger data collecting latency, especially for large-scale applications. In addition, combining the data collector and power transfer together has another inherent drawback, called the “doubly near–far” problem [20]; that is, the far-away sensors consume more energy in the UL and harvest less energy in the DL as a result of the distance-dependent power loss. To tackle these problems, a separately located power charger and data collector are considered as a more flexible way to balance the energy and information transmissions [21,22].

In conventional battery-powered WSNs, data-gathering techniques have been widely investigated in the last decade. In such works, as a result of uneven energy depletion with the distance to the predetermined sink, how to extend the network lifetime by designing a routing tree has attracted much attention. Uniform sensor deployment, resulting in the network lifetime decreasing by the sensors at the first hop from the sink, was first discovered in [23], and is also known as the “energy hole” problem. The authors in [24,25,26] have proposed several approaches to mitigate this problem. In [24], the authors propose an analytic model to estimate the entire network lifetime from network topology. This method can also be used to determine the boundary of the energy hole in a data-gathering WSN. In [25], the authors present a heuristic relay selection algorithm to balance the whole energy consumption, and a routing scheme based on maximum residual energy is proposed to mitigate the energy hole problem during data gathering. The authors in [26] investigated the energy hole problem and designed guidelines for maximizing the lifetime and avoiding energy holes in sensor networks with a non-uniform distribution. However, it should be noted that there exists a major difference between battery-based WSNs and WRSNs. In battery-based WSNs, one common objective is to minimize the total energy consumption of those sensors near the sink node, thus prolonging the network lifetime. However, such an energy balance-orientated design is not necessarily optimal for WRSNs, because of the fact that a high power consumption of any sensors in any location can now be replenished by means of WPT via the mobile chargers. This clear difference indicates that the routing techniques in WRSNs should be redesigned to fully take advantage of WPT. Unlike existing works, the aim of this work is to design a joint energy replenishment and routing mechanism for WRSNs. Specifically, given the velocity of the mobile charger, we aim to find the best traveling path corresponding with the optimal routing tree. In this way, we mitigate the gap between the heterogeneous energy consumption among the routing tree and the charging efficiency. To the best of our knowledge, this is the first study on the joint optimization for both energy charging and routing. The main contributions of this paper are summarized as follows:

- We present a novel joint optimization model including both charging efficiency and routing, rather than the typical charging optimization problem considering only the predefined data-gathering route.
- We propose a genetic algorithm (GA)-based optimization framework to find the optimal routing tree, in which the specific many-to-one routing tree is coded as an individual for evolution. We design an efficient individual encoding scheme and effective constraints handling mechanisms to achieve quick convergence.
- We then propose a heuristic algorithm to find the optimal resident locations with the given routing tree. By calculating the minimum moving distance and total charging time to evaluate the fitness of each individual, the evolution process of the GA is thus guided.
- We evaluate the proposed algorithms with extensive simulations and study the impact of multiple environmental factors, including the number of sensors and the types of routing tree. Our simulation results have showed that our proposed algorithm achieves a substantial improvement compared with the predefined route.

The remainder of this paper is organized as follows. In Section 2, we present the system model and problem formulation. Section 3 proposes our heuristic algorithm and the GA-based algorithm with computational complexities analysis. Section 4 presents numerical results, and Section 5 highlights our conclusions.

## 2. System Model and Problem Formulation

### 2.1. System Model

We consider a WRSN consisting of a single sink node and *N* sensor nodes, which periodically generate data with a different rate. The sensor nodes are labeled as N={1,…,N}. Without loss of generality, each node n∈N in the network has an initial energy $E_{i}^{n}$, a fixed communication radius $R_{c}$ and generates sensing data at a rate $r_{i}$. After deployment, we assume all the sensors can be accurately localized via existing localization algorithms, either centralized [27] or distributed [28]. The entire network can be represented as a directed topology graph G(N,E), where $e_{ij}\in E$ if $d_{ij}\leq R_{c}$, and $d_{ij}$ represents the distance between nodes *i* and *j* (i,j∈N,i≠j). We define the node’s dead state by its residual energy being below $E_{min}$. In this state, although it can still sense data, it cannot send its sensing data to the sink node either directly or indirectly via relay nodes, because of the energy constraint.

### 2.2. Routing Constraints

We assume each sensor node has the same sensing rate $\lambda_{n}$(n∈N) and define the real variable $f_{ij}^{n}$ (and $f_{ji}^{n}$) as the data flow traveling over link $e_{ij}$ (and over $e_{ji}$ ) for node *n*, where $e_{ij},e_{ji}\in E$. We have the following constraints when designing the routing tree:

For the source node *n*, we have

(1) $$\sum_{e_{ij}\in E}f_{ij}^{n}=\lambda_{n}\left(i=n\right)$$

For node *i*, as an intermediate relay node for sensor *n*, that is, i≠n,i≠Sink, we have

(2) $$\sum_{e_{ki}\in E}^{k\neq Sink}f_{ki}^{n}=\sum_{e_{ij}\in E}^{j\neq n}f_{ij}^{n}\left(i\neq n,Sink,i\in N\right)$$

For node *n*, as the sink node of sensor *n*, we have

(3) $$\sum_{e_{ji}\in E}f_{ji}^{n}=\lambda_{n}\left(i=Sink\right)$$

On the basis of the above definition, the energy consumption of each sensor *i* can be calculated as

(4) $$P_{i}=C_{i}^{r}D_{i}^{r}+\sum_{j\in N}C_{ij}^{t}D_{ij}^{t}$$

where $C_{i}^{r}$ is the energy consumption per receiving data rate of node *i*, $D_{i}^{r}$ is the total received rate of node *i*, $C_{ij}^{t}$ is the energy consumption per transmitting data rate from node *i* to *j*, and $D_{ij}^{t}$ is the total transmitted rate from node *i* to *j*. In Equation (4), $C_{ij}^{t}$, $D_{i}^{r}$ and $D_{ij}^{t}$ are given as

(5) $$D_{i}^{r}=\sum_{n\in N}\sum_{k\in N\backslash i,n}f_{ki}^{n}$$

(6) $$D_{ij}^{t}=\sum_{n\in N}f_{ij}^{n}$$

and

(7) $$C_{ij}^{t}=\beta_{1}+\beta_{2}d_{ij}^{\alpha }$$

where $\beta_{1}$ is a distance-independent constant term, $\beta_{2}$ is a coefficient of the distance-dependent term, and α is the path-loss index.

### 2.3. Energy Charging Cycle

We consider the scenario of a mobile charging vehicle periodically traveling inside the sensor network and charging each sensor node’s battery wirelessly.

To prolong the network lifetime, there exists a mobile charger *v* with a charging radius $R_{v}$ and an initial power $P_{v}$, which we assume can charge multiple sensors within its charging radius simultaneously. Similarly to [12,13,29], we assume the charging is only concerned with the distance and we ignore the environmental factors for simplicity. We thus define the charging rate of sensor *n* as

(8) $$U_{n}=\left\{\begin{array}{c} P_{v}u\left(d_{n,v}\right)\\ 0 \end{array}\right.\begin{array}{c} \mathrm{if}\;d_{n,v}\leq R_{v}\\ otherwise \end{array}$$

We assume this mobile charger charges the network with a fixed schedule *S* and a predefined traveling path *P* with a period of τ. The path *P* consists of a set of resident locations denoted as M={1,……M}. We denote the set of nodes covered by location *m* as $S_{m}$, which is given by

(9) $$S_{m}=\left\{n|d_{n,m}\leq R_{v},n\in N,m\in M\right\}$$

This should satisfy $N=S_{1}\cup S_{2}\cup \ldots \cup S_{M}$, which means that all sensors should be covered by all resident locations.

The charger starts from the first location and charges those nodes in $S_{1}$ with a time $\tau_{1}$. Then, it leaves the first location and moves to the second location with a traveling speed *v*. When it reaches the second location, it remains for time $\tau_{2}$ to charge the nodes in $S_{2}$. These steps are repeated until all the locations are visited once. After that, the charger returns to the first location to replace its battery and ready itself for the next charging tour. Thus, the cycle time τ can be written as

(10) $$\tau =\sum_{m\in M}\tau_{m}+\left(\sum_{m=1}^{M-1}\frac{d_{m,m+1}}{v}+\frac{d_{M,1}}{v}\right)+\tau_{vac}$$

where the first term is the time for charging, the second term is the time for moving, and the third term is the rest time.

For the resident location m∈M, the charging time $\tau_{m}$ depends on the charger’s residual energy and the node’s energy consumption rate. In order to ensure that all the nodes are still alive, the amount of charged energy for sensor $i\in S_{m}$ must be greater that the amount of consumed energy during the next cycle τ, which is expressed as

(11) $$\tau P_{i}\leq \tau_{m}P_{m}\;,\forall i\in S_{m}$$

where $P_{m}$ denotes the charger’s residual energy when it reaches the resident location *m*, which is given as

(12) $$P_{m}=P_{v}-\sum_{q=1}^{m-1}\sum_{n\in S_{q}}\tau_{q}P_{n}-\rho \sum_{q=1}^{m-1}d_{m,m+1}$$

**Figure 1:**
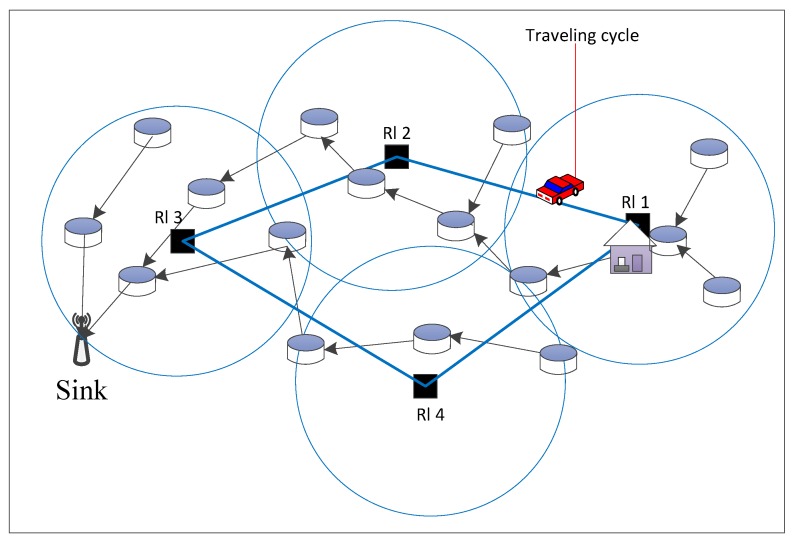
A mobile charger periodically visits each resident location once and charges those nodes in its charging radius.

Here, the second term is the consumed energy for the charging nodes from set $S_{1}$ to $S_{m-1}$, the third term is the consumed energy for moving, and ρ is the energy consumption for a moving unit of meters. One example of the charging model is illustrated in Figure 1 with 16 sensors and 4 resident locations. The mobile periodically visits each resident location and charges those sensors in its charging radius. How to find the optimal resident locations and the optimal routing tree to achieve the optimal charging efficiency is discussed in the next section.

### 2.4. Problem Formulation

In this section, we formulate our joint model, including both the charging efficiency and routing optimization.

**Charging efficiency problem**: Charging efficiency is conventionally regarded as the ratio between the working time and charging schedule. On the basis of this criterion, the objective of this problem is to maximize the charging efficiency for the networks. This can be achieved by searching the optimal resident locations, the optimal routing tree and the optimal traveling path. This problem is formulated as

(13) $$\begin{array}{c} \max\frac{\tau_{vac}}{\tau }\\ s.t.(1)∼(12) \end{array}$$

We note that the constraints of Equations (1)–(7) are the routing constraints, whereas the constraints of Equations (8)–(12) are the charging constraints. Instinctively, both of these two optimization problems are in the form of a non-linear programming (NLP) problem, which are generally non-deterministic polynomial-time hardness (NP-hard) and cannot be solved by traditional optimization methods [30]. In the next section, we develop the bio-inspired GA to solve these two optimization problems.

## 3. Optimization Algorithms

In this section, we propose two optimization algorithms to solve the joint charging and routing optimization problem. In both algorithms, we divide the optimization into two problems: the routing and the optimal charging. First, we propose the heuristic algorithm for optimal charging with a predefined routing tree, and then, the routing and charging are optimized in a combined manner. In this algorithm, the optimal tree is obtained by a GA, and the heuristic algorithm is used to evaluate the fitness of each individual in the GA and guide the evolution process.

### 3.1. Heuristic Algorithm for Optimal Charging

In this subsection, We assume the routing tree can be predetermined by some existing approaches, such as the Dijkstra routing algorithm or the minimum spanning tree (MST) routing algorithm. The optimization model can be transformed as the following:

(14) $$\begin{array}{c} \max\frac{\tau_{vac}}{\tau }\\ s.t.(8)∼(12) \end{array}$$

From the description of Equation (14), we know that the optimal charging efficiency is related to the resident locations M and the total moving distance $\sum_{m=1}^{M-1}\frac{d_{m,m+1}}{v}+\frac{d_{M,1}}{v}$. This total moving distance should be the shortest Hamiltonian cycle among all the resident locations. Otherwise, more time is spent on traveling. In addition, according to Equation (11), as a result of the lower left power, more time is needed to charge the sensors. Thus, the optimal charging efficiency is only related to the resident locations. According to Equations (8)–(12), these resident locations should cover all the sensors within its charging radius. Additionally, the Hamiltonian cycle among these locations should be the smallest. To satisfy these requirements, we therefore propose a heuristic algorithm shown in Algorithm 1 to find the optimal resident locations with the predefined routing tree. In our algorithm, we first introduce the following two definitions:

**Definition** **1.** *If a node has no neighbor nodes within a circle of radius $2R_{v}$, this node is defined as an isolated node.*

**Definition** **2.** *If a node has neighbor nodes within a circle of radius $2R_{v}$, this node is defined as a non-isolated node.*

We design the following two strategies to select the resident location for nodes of a different type. The first strategy is for isolated nodes. We first line an isolated node with the previous selected resident location; then, the location along this line and with a distance of $R_{v}$ to that isolated node is selected as the current resident location. The second strategy is for non-isolated nodes. We first select a node with the maximum distance to this non-isolated node within a distance $2R_{v}$. After that, the midpoint between these two nodes is selected as the resident location.

**Algorithm 1:** Heuristic algorithm for resident location selection
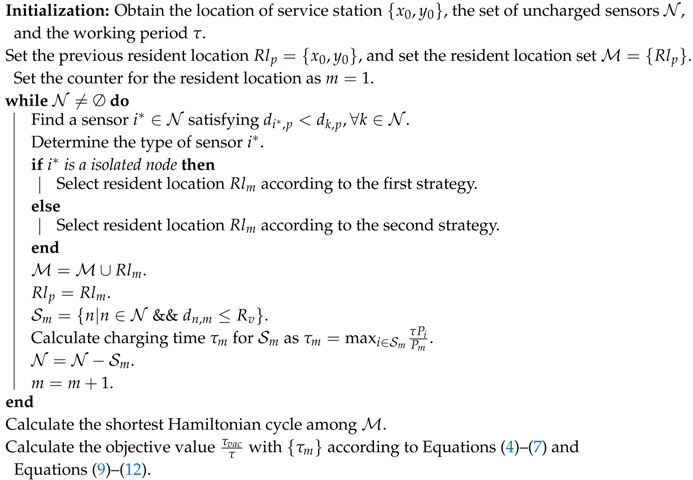


**Figure 2:**
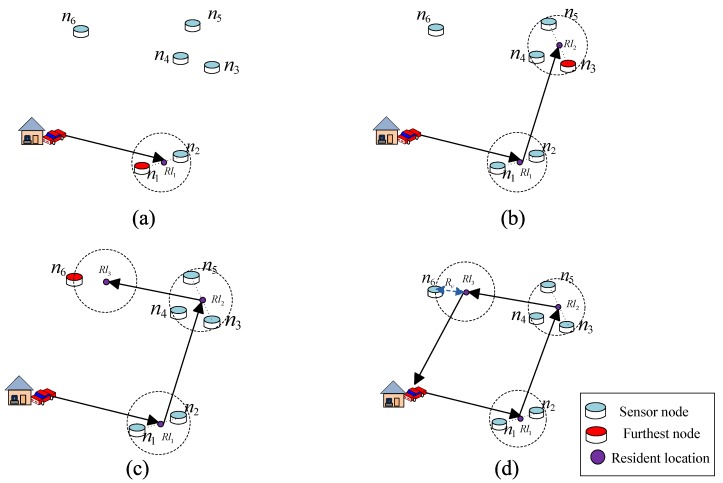
Illustration of how to select resident location.

As an example, Figure 2 shows how to select the resident location for different types of nodes. First, as shown in Figure 2a, node $n_{1}$ is selected as the first node near the service station, by judging its type as non-isolated. Node $n_{2}$ is selected, for the reason that $n_{2}$ has the maximum distance to $n_{1}$ within a distance $2R_{v}$. After that, the location in the middle of $n_{1}$ and $n_{2}$ is chosen and denoted as the first resident location $Rl_{1}$. Similarly to Figure 2a, we further select the second resident location $Rl_{2}$. Because node $n_{6}$ is an isolated node, $Rl_{3}$ is selected along the line between $Rl_{2}$ and $n_{6}$. Additionally, it has a distance of $R_{v}$ to $n_{6}$, as shown in Figure 2c. We therefore give the description of our heuristic algorithm in Algorithm 1 according to the following. In this algorithm, we first select these resident locations to cover all the sensors and calculate the minimum moving distance with the shortest Hamiltonian cycle. Then, with the energy consumption model defined by Equations (5)–(7), and with the charging constraints of Equations (9)–(12), the maximum charging efficiency is obtained. Here we assume that the mobile charger can visit any locations of the target area for simplicity. In fact, it is possible that some locations are hard to arrive at, because of the environmental factors. In this case, the heuristic algorithm should take these environmental factors into account. Additionally, the shortest Hamiltonian cycle among all the resident locations should be redesigned, for the reason that some resident locations cannot be directly connected.

### 3.2. Joint Optimization of Routing and Charging

In order to find the best routing tree corresponding to the selected resident location, a straightforward solution is to conduct an exhaustive search by constructing all feasible routing trees. This approach, however, is infeasible for networks with a larger number of sensor nodes. The heuristic algorithm is based on decomposition. However, this approach may be suboptimal because of the fact that the routing tree and charging efficiency interact with each other and the routing and charging should be optimized in a compact form. Therefore, we apply the GA to integrate these two schemes to achieve the interaction between the charging efficiency and the routing tree.

By simulating the process of evolution in the natural system, the GA can be considered as an adaptive heuristic search algorithm that is very suitable for providing a robust, near optimal solution for many real world NP-hard problems, and it is also widely applied for the performance optimization of WSNs, such as in network coverage control for WSNs [31,32,33], the scheduling problem [34,35], the optimal sensor deployment problem [36,37], and topology control [38]. This bio-inspired algorithm imitates the natural evolution of biological organisms to provide a robust, near-optimal solution for various problems. The GA is inherently an evolutionary process that involves individual encoding, selection, crossover, mutation, and replacement operations [39]. 

#### 3.2.1. Individual Encoding

The GA cannot deal with the solutions of the optimization problem directly. The solutions need to be represented as chromosomes in terms of the data structure. In our optimization problems, a tree-based encoding scheme is proposed to represent the potential solutions.

**Figure 3:**
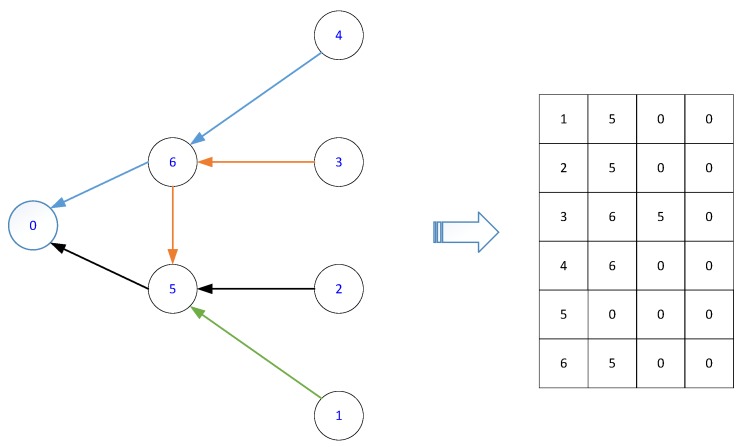
Individual encoding scheme.

In our scheme, the routing paths of all the sensors to the sink node are randomly generated, to thus explore the genetic diversity. For each sensor *i*, its first forward node $i_{f}^{1}$ is randomly selected from its neighboring set $N_{i}/i$. Then, we randomly select the second forward node $i_{f}^{2}$ from the neighboring set $N_{i_{f}^{1}}/\{i,i_{f}^{1}\}$. This step is repeated until the data is received by the sink node. In the above path construction, the sensors already in the routing path are excluded, thereby to re-enter the same node. We denote the path of sensor *i* as $P_{i}=\left\{i,i_{f}^{1},i_{f}^{2},\ldots ,sink\right\}$, and we can conclude that the maximum length of path $P_{i}$ is less than N−1 because the total number of sensors is *N*. After we have found routing paths for the sensors in N, these paths are further combined as a reverse many-to-one multicast routing tree ending at the only sink node. This reverse multicast tree is further mapped as a matrix $\Gamma_{N*N-1}$, where the *i*th row denotes the routing path of sensor *i*. Figure 3 illustrates an example of six routing paths and their chromosome representation, where the sink node is denoted as node 0. With this coding scheme, we first generate an initial population R with *R* matrices.

Additionally, considering that the routing tree obtained by Dijkstra’s algorithm is also a sub-optimal solution, we thus take it as a potential individual in the initial population. In this way, the initial population in our algorithm contains R−1 randomly generated individuals and one existing sub-optimal solution. By doing so, this initialization can converge much faster than that without exploiting the knowledge we already have. The population initialization procedures are described in Algorithm 2. We note that the initialization procedures can generate *R* different routing trees, and each routing tree satisfies those routing constraints defined by Equations (1)–(7).

**Algorithm 2:** Population initialization
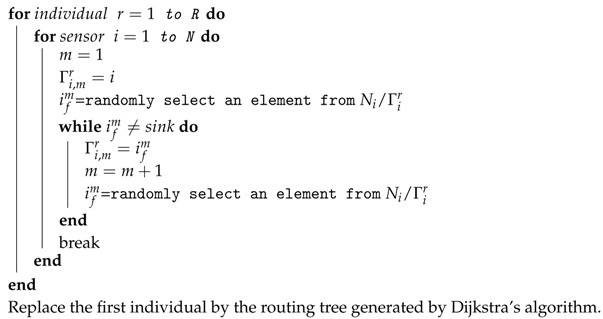


#### 3.2.2. Fitness Functions and Natural Selection

In the GA, the selection operation is applied to choose individuals to participate in reproduction, which has a significant impact on driving the search towards a promising trend and finding optimal solutions in a short time. We adopt the famous roulette wheel selection method to select the individual on the basis of its selection probability, which is proportional to its fitness function. The selection probability of the *r*th individual is defined as

(15) $$\begin{array}{c} q_{r}=\frac{f\left(r\right)}{\sum_{r\in R}f\left(r\right)} \end{array}$$

where f(r) is the fitness function of individual *r*. In this paper, the fitness value is defined as the charging efficiency obtained from Algorithm 1 with the given routing tree.

#### 3.2.3. Crossover and Mutation

**Figure 4:**
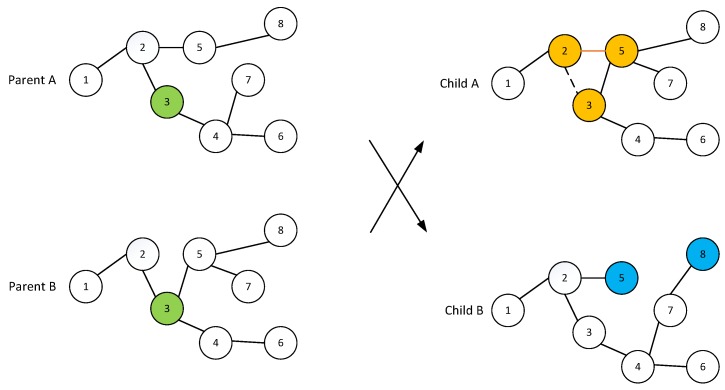
Tree-based crossover operation.

The crossover operation is used to mix the individuals to increase their fitness. Considering that the chromosomes are expressed by a tree data structure, a single-point crossover is designed to exchange a partial chromosome. For the two individuals selected to crossover, we first select common nodes between these two trees; then, one common node among them is randomly selected as the crossover point. After that, the subtrees rooted from this node are swapped to generate two new routing trees. We illustrate an example of single-point crossover and the individual repair operation in Figure 4, where the common node is node 11. The crossover between parent A and parent B is performed by switching the subtree rooted at node 3 with that of parent B. After the crossover, the new generated routing tree in child A has a routing loop of 2–3–5. As such, we repair this routing loop by randomly deleting link $e_{23}$. Additionally, nodes 5 and 8 are randomly linked to child B to ensure the feasibility.

In the mutation operation, the elements in both matrices of each individual are randomly altered to diversify the population after the crossover operation, which will pave the way towards global optima. In this paper, once a node (denoted as *n*) is selected as the mutation point with probability $q_{m}$, the mutation replaces the path *n* to the sink node by a randomly generated new path. It is observed that the designed genetic operation still generates routing trees, indicating that routing constraints are still satisfied.

#### 3.2.4. Replacement

After generating a new population through the crossover and mutation operators, an elitist model-based replacement is employed to update a certain number of individuals in the old population with the new generated individuals. The low-quality individuals with low fitness values in the parental population are replaced by their children in the next generation.

Now, we have designed the key components of the GA operation, which are the individual encoding, population initialization, selection, crossover, mutation, and replacement operation. The joint optimization of routing and charging based on the GA is depicted in Algorithm 3, where *G* is the given number of generations, *R* is the population size, $q_{c}$ is the crossover probability, and $q_{m}$ is the mutation probability. 

**Algorithm 3:** Joint optimization based on genetic algorithm
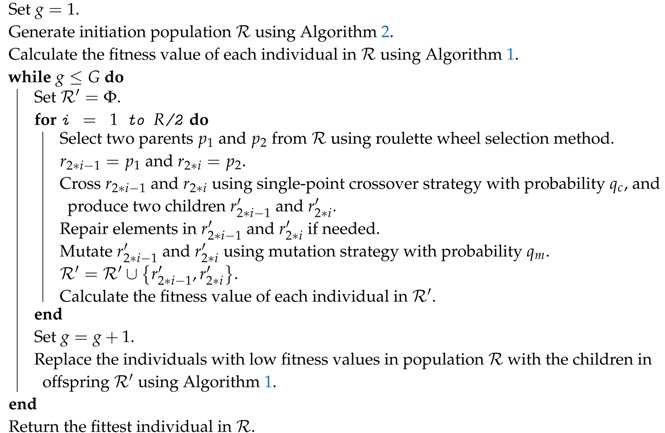


In the proposed GA-based optimization, the computational complexity is dominated by the complexity in evaluating the fitness of Algorithm 1, which has to be evaluated *R* times in each iteration. The time complexity for calculating the fitness function of the resident locations is O(N) within an iteration. Aside from this, a GA-based approach also depends on other factors, which are difficult to clearly enumerate, such as strategies to generate a new population, and the tolerance allowable for cumulative changes in the fitness values [40]. Excluding these parameters, the total complexity of our algorithm is $O(G\left(NR+R^{2}\right))$. Similarly to that in [12,19,41], our algorithms are performed in an offline manner. We assume the algorithm is executed at the sink node until the optimal routing tree and charging schedule are obtained. After that, the obtained routing tree is sent back to all the sensors via broadcasting, and the resident locations and traveling path are sent to the mobile charger. To run our algorithm, the sink node needs to collect the ID and location of each sensor. After receiving the results, one sensor can quickly find its forward sensor by only exchanging its IDs with its neighbors.

## 4. Numerical Results

In this section, we provide numerical results to illustrate the performance of our proposed algorithm. We consider a WRSN with a fixed sink node, and no more than 80 sensor nodes are randomly deployed in a square area with size 500 × 500. We assume the sink node is located in the center of the area, the service station is located at the corner, and the moving speed of the mobile charger is 5 m/s. The details of these parameters are summarized in Table 1 unless otherwise specified. The corresponding simulations were implemented in Matlab 7 using a laptop with an Intel (i5-4300) CPU. All the results were obtained by averaging over 100 simulations.

### 4.1. Convergence Behavior

In the GA, the convergence behavior is affected by many control parameters, such as the initial population, the mutation probability and the crossover mechanism. To the best of our knowledge, the conditions for GAs to converge have been proved only for binary encoding with Markov chain models [42]. However, for the GA algorithm with integer encoding, the convergence is still an open problem [43]. In this paper, rather than using an analytical approach, extensive simulations are employed to investigate the convergence issue. In our simulations, we set the maximum number of generations as 500. In fast, the number of generations depends on the number of individuals. For instance, more generations are needed for a greater number of sensors.

**Figure 5:**
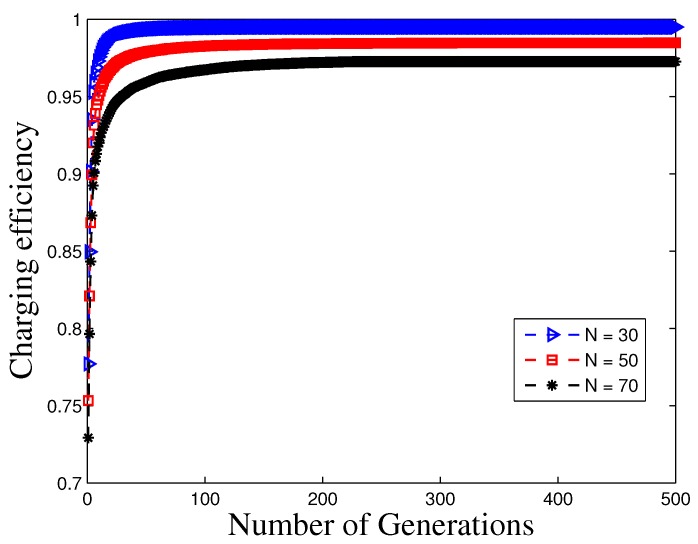
Convergence behavior versus different number of generations.

Figure 5 plots the convergence behavior of the charging efficiency with the number of generations, and we can observe that the algorithm converges after approximately 300 generations for various numbers of sensors. It takes 50 s to converge for N=70 sensors. This is sufficient for many applications. If we use a more powerful computer, it is expected that it can converge much faster. For our optimization problem, the charging efficiency with a random generated routing tree at the initialization is 72.9%, while the final obtained value after optimization with the GA is 99.5%, which showcases that the GA achieves a nearly 50% greater charging efficiency compared with that of the random routing tree. This also indicates that it is important to design a joint optimization including both routing and charging. Additionally, it is revealed that the converge speed can be substantially increased with a reduced number of sensors.

### 4.2. Total Traveling Distance

**Figure 6:**
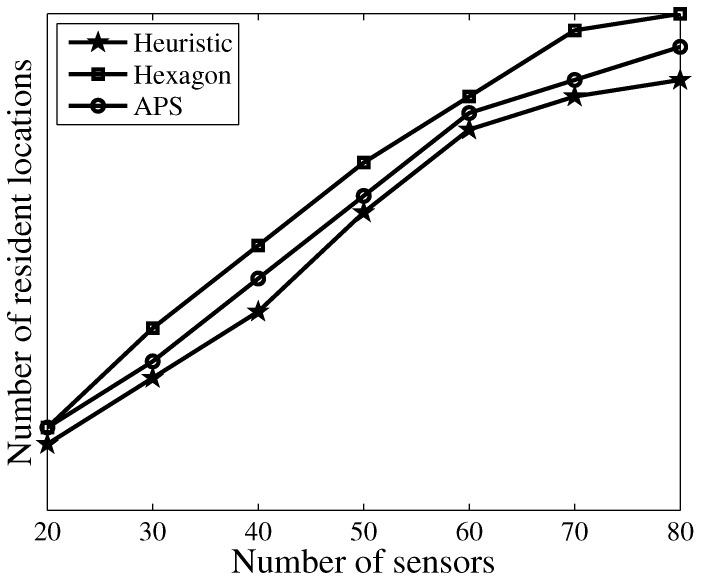
Number of resident locations versus different number of sensors.

**Figure 7:**
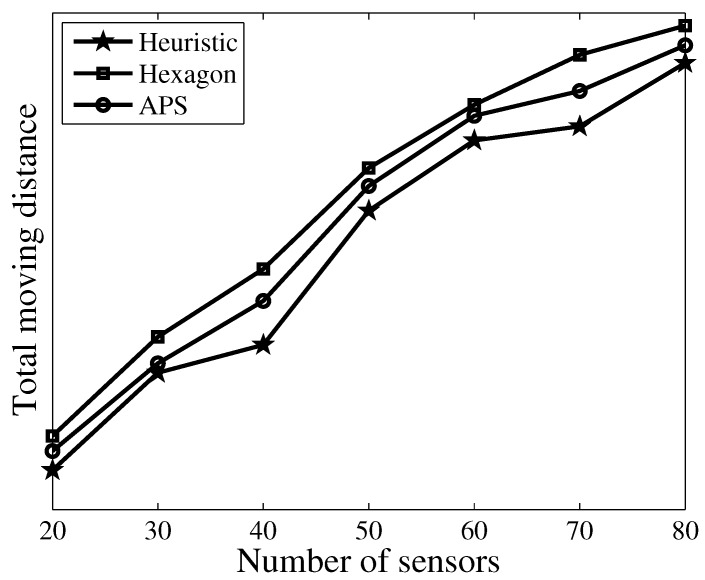
Total moving distance versus different number of sensors.

Figure 6 plots the number of selected resident locations versus a different number of sensors. We also compare this with using the hexagon algorithm in [12] and the anchor point selection (APS) algorithm proposed in [17]. We observe that the number of resident locations increases by increasing the number of sensors. More importantly, our algorithm obtains fewer resident locations than the other two algorithms. This can be explained by the fact that resident locations with the hexagon algorithm are selected in the adjacent hexagons one by one, and the number of sensors covered by each resident location is random. In APS, the resident location is always selected as the location of the sensor node, while in our approach, we always aim to charge the largest number of sensors in one resident location; therefore, few resident locations are needed to cover all the sensors. Figure 7 further plots the total traveling distance versus a different number of sensors. As expected, we observe that our approach obtains fewer traveling distances than the other two algorithms. Another observation is that the total traveling distance increases with an increasing number of sensors.

### 4.3. Routing

**Figure 8:**
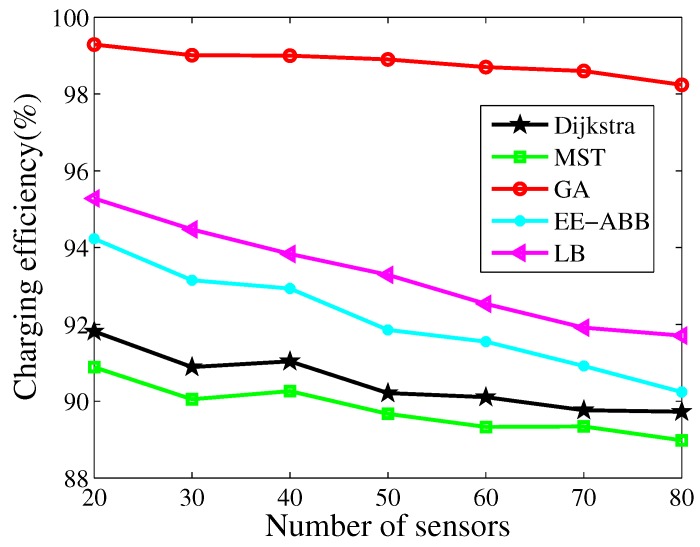
Comparison with the charging efficiency.

**Figure 9:**
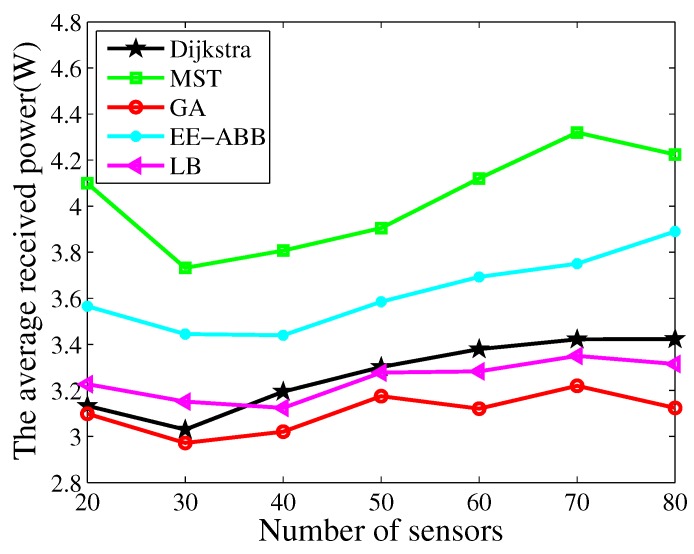
Comparison with the average received power.

Figure 8 plots the comparison of the charging efficiency with different algorithms. Here, LB is the low latency based routing algorithm proposed in [25], where the sensor with the maximum residual energy in the next forward ring is selected as the forwarder. EE-ABB is an energy-efficient routing protocol based on an artificial bee colony algorithm [44], where an intelligent clustering algorithm is applied to improve the performance of LEACH (low-energy adaptive clustering hierarchy protocol) [45]. It is observed that the charging efficiency decreases with an increasing number of sensor nodes. We can also observe that our algorithm achieves the best performance compared with other algorithms. Figure 9 plots the average received power comparison of these algorithms. We can observe the same trend as in Figure 8. Additionally, we can observe that the LB algorithm achieves a better performance than the clustering algorithm, which is mainly because of the higher energy consumption of the cluster node, and more charging time being spent on those cluster nodes. We can also observe that the substantial improvement in the charging efficiency is achieved by jointly optimizing both charging and routing in our algorithm, which also demonstrates the effectiveness of our algorithm.

## 5. Conclusions

In this paper, we have presented the joint optimization model, including both the charging efficiency and routing for WRSNs. We have developed a heuristic algorithm to obtain the optimal charging efficiency with the predefined routing tree. We have also proposed an efficient GA-based algorithm for the joint optimization of routing efficiency and charging efficiency. The number of resident locations and total traveling distance can be improved by choosing the optimal resident locations with the proposed heuristic algorithm. Jointly optimizing both charging and routing can further improve the charging efficiency compared with the existing routing algorithms. 

## Figures and Tables

**Table 1:** Simulation parameters.

Parameters	Value
The initial energy of sensor *n*, $E_{i}^{n}$	10,800 J
The minimum energy for working, $E_{min}$	540 J
The mobile speed, *V*	5 m/s
The full charging ratio, $u_{Full}$	5 W
The minimum charging ratio, $U_{min}$	1 J
The charging radius, $R_{v}$	2.7 m
The sensing ratio of node, *n* $\lambda_{n}$	1∼10 kbps
The number of sensors, *N*	20∼80
Distance-independent constant term, $\beta_{1}$	$50\times 10^{-9}$ J/b
Coefficient of distance-dependent constant term, $\beta_{2}$	$0.0013\times 10^{-12}$ J/(bm${}^{4}$)
Pass-loss index , α	4
Energy consumption for receiving per data rate, $C_{i}^{r}$	$50\times 10^{-9}$ J/b
